# Penile carcinoma: a report of two cases treated by partial penectomy, its effects on quality of life and review of literature

**DOI:** 10.11604/pamj.2022.41.33.29970

**Published:** 2022-01-13

**Authors:** Kekeli Kodjo Adanu, Bernard Toboh, Evans Akpakli, Mary Monney, Isaac Asiedu, Maxwell Nyinah, Bright Wereh, Yaw Amoah, Matthew Kyei, James Edward Mensah

**Affiliations:** 1 Department of Urology, Korle Bu Teaching Hospital, Accra, Ghana,; 2 Department of Surgery, University of Health and Allied Sciences, Ho, Ghana

**Keywords:** Penile cancer, partial penectomy, quality of life, Ghana, case report

## Abstract

Penile cancer is a rare malignancy worldwide except in parts of Africa, Asia and Latin America where higher incidences have been reported. The disease leads to serious physical disfigurement of the male genitalia which can have debilitating consequences, thus it may alter micturition patterns and impair penetrative sexual intercourse. The lack of cancer registries and epidemiological surveillance programs in Ghana makes estimation of the prevalence in Ghana difficult hence to advance the course of knowledge, awareness and prevention of penile cancers, it is imperative that such cases are brought to the fore and discussed. We report two cases of penile cancer that had partial penectomy and inguinal lymphadenectomy at the Korle Bu Teaching Hospital. Clinical findings and intervention of these reported cases highlight the management process and it further assessed the psychological impact of intervention. The two patients presented to our outpatient department with penile lesions which were confirmed to be penile cancer. The first patient presented with a 30 year history with recurrent ulceration while the other presented with just 1 (one) year history of penile lesion. Both patients required partial penectomy and inguinal lymphadenopathy in the treatment of their condition. The major risk factors as reported in this case study, include uncircumcision, previous treatment for sexually transmitted infections, multiple sexual partners and smoking. Treatment is associated with reduction in sexual function although quality of life may remain satisfactory. Strong family and psychological support are key cornerstones for good treatment outcomes.

## Introduction

Penile malignancy is rare worldwide, with developed countries in Europe and America reporting incidences of less than 1 case per 100,000 men annually [1,2]. However, the situation is dissimilar in developing countries of sub-Saharan Africa, parts of Asia and Latin America where relatively high incidence rates have been reported. Romania, Brazil, Uganda, Zimbabwe and Malawi have reported incidences higher than 2/100,000 men over the decades [1]. An institutional study in Ghana, reported a rate of 1.8% of all genitourinary cancers [3]. Between 2015 and 2020, four cases of penile cancer were recorded at the Korle Bu Teaching Hospital. Recent studies have shown a steady decline in the incidence rates presumably as a result of the improvement in socio-economic conditions in many developing countries [1]. This cancer leads to serious physical disfigurement of the male genitalia which can have debilitating consequences, thus it may alter micturition patterns and impair penetrative sexual intercourse [4].

In conservative societies where masculinity is related to the phallus, men affected become psychological distressed, impinging their self-esteem. Most cases present between 50 and 70 years of age, with majority of cases in sub-Saharan Africa presenting in the advanced stages of the disease, associated with a five-year survival rate of 50%. When pelvic lymph nodes are involved, survival may approach 0% [2,5]. Most cases presents with a lesion on the glans (48%), followed by the prepuce (21%), on the prepuce and glans (15%), coronal sulcus (6%) and penile shaft (<2%). The clinical presentation varies, with the initial symptoms being an induration or erythema, progressing to blebs, infiltrative and then ulcerating lesions. Associated with these, are constitutional symptoms of penile discharge, pruritus, bleeding, pain and foul smell [5].

The lack of cancer registries and epidemiological surveillance programs in Ghana makes estimation of the prevalence in Ghana difficult, hence to advance the course of knowledge, awareness and prevention of penile cancers, it is imperative that such cases are brought to the fore and discussed. We report two cases of penile cancer that had partial penectomy and inguinal lymphadenectomy at the Korle Bu Teaching Hospital and also assessed the quality of life and effect of cancer on sexual health post-surgery. These are men in the prime of their lives who may be eternally affected by the disfigurement of surgery and the psychological implications of same. Early detection and prompt management may therefore be a key in reducing the psychological burden on families. These cases reported highlights the management process and it further assessed the psychological impact of intervention. It is anticipated that these discussions would serve as the foundation for a more comprehensive study on the incidence and sub types of penile cancers in Ghana.

## Patient and observation

### Case 1

**Patient information and history:** a 56-year-old man presented for evaluation of a penile ulcer present for 30 years which started as a papulo-vesicular lesion on the dorsum of the glans penis. The lesion ruptured spontaneously and had periods of healing and recurrent ulceration until 3 years before presentation when it became persistent without healing. He was uncircumcised until 16 years of age when he underwent circumcision due to the recurrent ulceration. Apart from being recurrent, the ulcer had also increased in size since it was first noticed. He had curettage and biopsy of the ulcer at a peripheral facility in 2019 which revealed the presence of condyloma acuminatum and was referred for further care. Our patient had 9 lifetime sexual partners and takes alcohol occasionally. He admitted to a history of lower urinary tract symptoms (dysuria and frequency), however, denied any history of prior urethral discharge, anal sexual activity and smoking. He was a farmer.

**Physical examination and clinical findings:** physical examination was significant for a fungating ulcer, 7 cm x 7 cm, with raised edges, nodular floor which occupied the distal two-thirds of the penis with lateral deviation of the urethral meatus. Inguinal lymph nodes were palpable bilaterally-non tender and discrete. Scrotum examination was normal.

**Diagnostic assessment:** a second incisional biopsy confirmed a well differentiated squamous cell carcinoma and abdominopelvic computed tomography (CT) scan showed an ill-defined mixed density inhomogeneously enhancing mass involving the penis. The mass was associated with multiple enlarged lymph nodes in both inguinal regions (two on the right and four on the left). The largest of these lesions on the right and left sides measure 17 mm and 13 mm respectively. The internal organs were normal and no ascites or any abnormal intra-abdominal masses were noticed.

**Figure 1:**
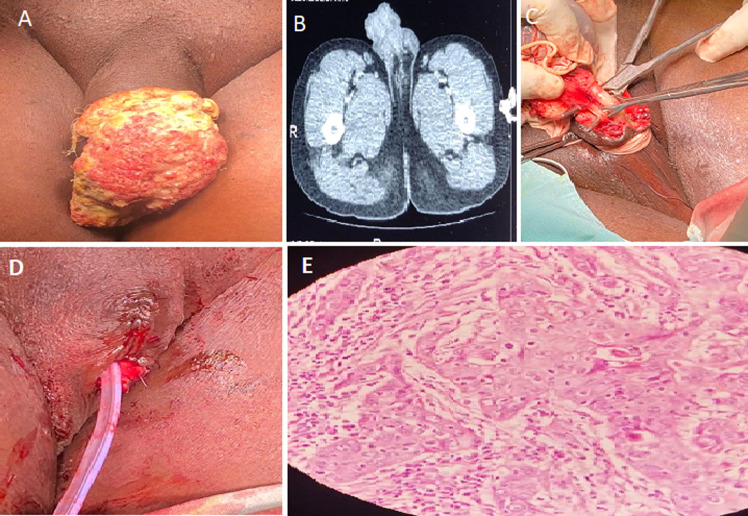
A) 7 cm x 7 cm erythematous fungating ulcerative lesion with nodular floor and raised edges; B) sagittal view of abdominopelvic CT scan showing penile tumor and multiple enlarged lymph nodes in inguinal region; C) dissection of urethra and refashioning of penile stump; D) closure of defect and urethral catheter left in-situ; E) close up view of histological specimen showing nuclear pleomorphism and mitoses (hematoxylin & eosin stains x400)

**Therapeutic interventions:** based on these, the patient was counselled and prepared for a partial penectomy with inguinal lymphadenectomy. The surgery was performed under general anaesthesia and aseptic conditions. A tourniquet was applied at the base of the penis and a circumferential incision made approximately 1.5 cm below the lesion. The incision was deepened through dartos, bucks fascia to the tunica albuginea. The superficial penile vessels and deep dorsal neurovascular bundles were ligated using vicryl 2.0 suture and transected. Both corpora cavernosa were transected symmetrically and the urethra dissected out and also transected leaving a 1 cm urethral cuff. The urethra was spatulated dorsally and a neomeatus fashioned out by approximation to the dorsal corpus spongiosum. The defect was then repaired using linear closure. Through a right inguinal incision and via sharp and blunt dissection a superficial inguinal lymphadenectomy was achieved (Figure 1).

### Case 2

**Patient information and history:** the second patient was a 50-year-old man who first noticed a nodular lesion on the tip of the glans penis about 9 months prior to presentation. He initially resorted to self-medication but upon worsening of his condition, he visited a peripheral health facility where he was investigated for sexually transmitted infections, all of which were negative. A biopsy done confirmed an invasive squamous cell carcinoma hence referred on that basis. He admitted to a history of lower urinary tract symptoms (dysuria), penile pain, contact bleeding and previous treatment of gonorrhea and herpes simplex (20 years ago). He denied any urethral discharge on current presentation. He is single, has no children, had 11 lifetime sexual partners, smokes cigarettes (5 pack-years) and takes alcohol (hard liquor) quite frequently. He has no known chronic illness.

**Physical examination and clinical findings:** physical examination was significant for a well looking man, with good performance status (ECOG 0). Penile examination revealed a glans ulceration, 4 cm x 4 cm, with a nodular floor, minimal slough and no obvious discharge. The inguinal lymph nodes were palpable-discrete and non-tender.

**Diagnostic assessment:** an abdominopelvic CT scan noted multiple bilateral inguinal lymph nodes, more on the right than the left. The largest lymph node on the right measured 2.8 x 1.5 cm and on the left, 1.5 x 1.0 cm. There was a locally invasive mass around the glans and the foreskin of the penile shaft. The urethra was however not obstructed or distended.

**Figure 2:**
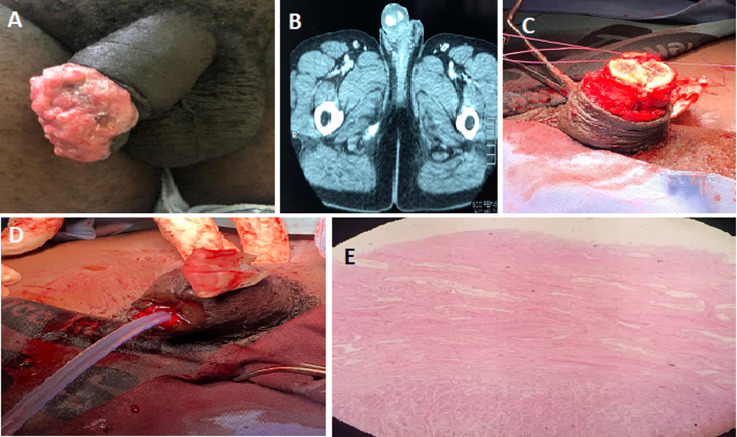
A) 4 cm x 4 cm fungating ulcerative lesion of the glans, nodular floor and raised edges; B) abdominopelvic CT scan of tumor with inguinal lymphadenopathy; C) repair of corpus cavernosa remnants; D) linear closure of defect with urethral catheter and wound drain left in situ; E) squamous cell carcinoma with tumor invading spongiosum with urethral urothelium at the top (hematoxylin & eosin stain x40)

**Therapeutic interventions:** similarly, he was counselled and prepared for a partial penectomy and inguinal lymphadenectomy. The main findings were a fungating ulcer (4 cm x 4 cm) involving the glans penis and the distal penile shaft; and discrete right inguinal lymph nodes. Histopathology reported a well-differentiated squamous cell carcinoma; with chronic inflammatory infiltrate. Tumor involves the spongiosum with perineural invasion but no lymphovascular invasion. The excised margins were free. One of nine lymph nodes had tumor deposits. The diagnosis of an invasive well differentiated squamous cell carcinoma was confirmed (pT2N1). Figure 2 depicts our findings. He was subsequently referred to the radio-oncology unit for chemoradiation.

### Follow up and outcomes

**Effects on quality of life:** in order to assess the psychological implications, the effects on the quality of life and sexual health of patients following treatment, we used the EORTC QLQ-C30 and the EORTC SHQ-C22 assessment tools. These are standardized and validated questionnaires designed by the European Organization for the Research and Treatment of Cancer (EORTC) to assess the health related quality of life of cancer patients. While the EORTC QLQ-C30 (C30) assesses the psychological implications and the quality of life, the EORTC SHQ-C22 (C22) focuses on the effects on sexual health. The C30 has physical, role, emotional, cognitive and social functioning as its main domains while assessing other factors such as fatigue, pain, difficulty in breathing and financial difficulties. The C22 had effects on sexual satisfaction, sexual pain and importance of sexual activity as its main domains. The questionnaires were administered a year post-surgery.

Sadly, one of our patients (case 1) died some months after surgery hence his data could not be collected. Nonetheless, having considered the rarity of the condition and the apparent paucity of information on the disease in sub-Saharan Africa, we deem it necessary to report on these findings. The surviving patient (case 2) achieved a physical functioning score of 80%, role functioning score of 83%, emotional score of 75% and a social functioning score of 67%. His lowest score was 50% for cognitive function. He admitted to experiencing fatigue about a third of the time (33%), vomited quite often (67% of the time) and occasionally experienced difficulty in breathing (33%). In terms of sexual function, he admitted to a considerable dip in sexual satisfaction (57%), experienced some pain during intercourse (11%) but had not noticed any decline in libido (100%). Table 1 and Table 2 illustrate aggregated scores for quality of life and sexual function.

**Table 1:** quality of life scores

Domain	Raw score (max score = 4)	Standardized score (%)
**Functional scales**		
Physical functioning	1.60	80.00
Role functioning	1.50	83.30
Emotional functioning	1.75	75.00
Cognitive functioning	2.50	50.00
Social functioning	2.00	66.67
**Symptom's scale**		
Fatigue	2.00	33.33
Nausea and vomiting	3.00	66.67
Pain	2.50	50.00
Dyspnoea	2.00	33.33
Insomnia	3.00	66.67
Appetite loss	3.00	66.67
Constipation	2.00	33.33
Diarrhoea	1.00	0.00
Financial difficulties	3.00	66.67

**Table 2:** sexual function scores

Domain	Raw scores (max score = 4)	Standardized score (%)
**Functional scales**		
Sexual satisfaction	2.71	57.00
Importance of sexual activity	2.00	33.33
Libido	1.00	0.00
Effect of treatment	2.00	66.67
Communication with professionals	1.00	0.00
Insecurity with partner	2.00	66.67
Confidence erection	3.00	66.67
Masculinity	2.00	66.67
**Symptom scales**		
Sexual pain	1.33	11.00
Worry incontinence	2.00	33.33
Fatigue	1.00	0.00

## Discussion

Although there are several sub-types, the preponderance of penile cancers are squamous cell carcinomas [6]. The pathogenesis still remains obscure even though several risk factors have been identified. It is thought that it may arise *de novo* or from a pre-existing penile intraepithelial neoplasia [5]. Among the host of risk factors, phimosis, the inability to retract the penile foreskin, in uncircumcised males and human papillomavirus (HPV) are the most significant. Phimosis is associated with poor hygiene with accumulation of smegma, and chronic inflammation of the penis. It has been linked to about 90% of penile cancers with lower incidences among the Jews, due to the high rates of circumcision in this ethnicity. These related events lead to the progress from penile intra-epithelial neoplasia to overt cancer. Neonatal circumcision is therefore thought to be protective against the disease [4,5]. HPV types 16 and 18 have also been widely associated with penile cancer. Heideman and his colleagues [7] concluded that HPV 16 is the main aetiological type involved in the pathogenesis of penile cancers. They performed an investigation on a large series of 83 patients to detect the presence and biologic activity of a spectrum of mucosal and cutaneous HPV sub-types and found a high viral load and gene expression of the HPV-16 in most tumors. Current data suggest that up to 50% of cases results from a step wise progression from HPV associated preneoplastic lesions [8].

In addition to the aforementioned risk factors, smoking, prominent sexual history and exposure to ultraviolet radiation are also implicated [4,8]. The risk of smoking is said to be dependent on smoking habits with chain smokers being at a higher risk. The exact mechanism is unknown but could be related to the accumulation of nitrosamines in genital secretions [8]. The number of sexual partners has also been directly linked to the disease. Men with more than thirty lifetime partners have a 3 fold increased risk compared to men with less and a history of condyloma accuminatum is associated with a 4-5 times increased risk [8]. Lichen sclerosis, ultraviolet therapy, bowens disease, erythroplasia of queyrat, penile trauma, low socio-economic status and poor education have also been associated with penile cancers by a number of studies [2,4,8]. The concept of low socio-economic status may have a telling impact on geographic distribution of penile cancers. In the last decades of the twentieth century, higher incidences were recorded in least developed countries compared to the more developed nations [1]. However, with the decline in poverty and rapid urbanization, countries such as Uganda have noted a steady decline in incidence rates to 3 to 4/100,000 men from a high of 6.3/100,000 in the earlier decades of the twentieth century [1,2].

Treatment of penile cancers depends on the stage at presentation. Squamous cell carcinomas (SCC) makes up 95% of all penile malignancies, and about half of these originate from the keratinized epithelium of the glans or the prepuce. There are several sub-types of penile SCC, prominent among these are the verrucous, basaloid, warty-type and the clear cell carcinomas [5,9]. The inguinal lymph nodes are usually the first site of metastases followed by the pelvic lymph nodes and rarely, the retroperitoneal nodes. Late metastases may occur to the bones, lungs and the liver. The ability or the lack thereof to palpate lymph nodes is not an accurate indication for inguinal lymphadenectomy. Inguinal nodes are palpated in just over 60% of cases [6]. It is therefore essential that imaging such as abdomino-pelvic CT scans, magnetic resonance imaging (MRI) and other diagnostic parameters are employed to accurately stage and manage penile cancers. The 2016 tumor nymph metastasis (TNM) classification for penile cancers and the European Association of Urology guideline [10] on penile cancer was adapted in the management of our patients while taking due cognizance of the available local resources.

The aim of primary tumor removal is complete tumor excision with preservation of the penile organ as much as possible without compromising tumor control. Penile preservation appears to be more beneficial in functional and cosmetic outcomes to partial or total penectomy. The treatment of small and localized penile cancers includes external beam radiation therapy (EBRT), brachytherapy, excisional biopsy and laser therapy. For superficial non-invasive disease, treatment options comprise topical chemotherapy using 5 fluouro-uracil and laser ablation; in recurrent cases, glansectomy, partial or total penectomy are indicated. For invasive disease confined to the glans (T1/T2); local excision, partial or total glansectomy are suitable surgical options. Other modalities such as EBRT, brachytherapy and laser ablation may be employed but with due cognizance of the tumor size, histology, stage and grade [10]. The main treatment modalities for invasive penile cancers (T2-T4 disease) include total glansectomy, partial or total penectomy. In most cases of invasive penile cancers, preservation of the penile shaft is practically impossible [9-11]. The development of metastatic lesions is dependent on the lymphatic drainage from the penis. The inguinal lymph nodes are first affected followed by the pelvic and in some cases the retroperitoneal lymph nodes. Pelvic nodal disease does not usually occur in the absence of ipsilateral lymph node involvement. Radical lymphadenectomy seems to be the treatment of choice, although combination therapy of surgery and chemotherapy is also advocated [10].

Moh´s micrographic microsurgery is an effective tool for management of invasive penile cancers. It involves resecting the primary tumor under magnification to achieve the smallest negative margin in order to preserve as much penile tissue as possible. A study consisting of 33 patients treated using this technique reported a cure rate of 79% with one recurrence and one cancer specific mortality [11]. The unavailability of this equipment in developing countries and the advanced stage at which most of these tumors present makes it practically impossible to use this technique in sub-Saharan Africa. Regardless of the stage, tumors with high grade features are associated with a poorer prognosis and higher likelihood of metastases. The most important prognostic factor is the presence of inguinal lymph nodes [10,11].

The two cases of penile cancer presented occurred in men between the ages of 50-60, which is consistent with the peak age for the development of penile cancers [2,5]. The most significant risk factors gleaned in these two cases include uncircumcision, multiple sexual partners, previous treatment for sexually transmitted diseases and a strong smoking history. Uncircumcision which may consequently cause phimosis has been strongly associated with the development of penile cancers. Morrison [4] opines that circumcision reduces the risk of infection of oncogenic-serotypes of HPV which serve as precursors in penile carcinogenesis. Uncircumcision as a risk in sub-Saharan Africa remains variable. In some countries such as Ghana, circumcision is religiously practiced, while in others such as Kenya and Tanzania, most men are uncircumcised [2]. In a study conducted in Uganda, there was a higher rate of HPV in swabs taken in uncircumcised males compared to swabs taken a year after circumcision [12]. The HPV-16 has been isolated as a carcinogen in a subset of penile malignancies with its detection rates varying from 17% to 82% in several series [7,8]. Evidence of the linkage between multiple sexual partners and penile cancers has been clearly adduced in several studies.

Men with more than thirty lifetime sexual partners are at a threefold increased risk for penile cancer compared to those with less [8]. Morrison [4] also averred that sexual promiscuity could be an important risk factor. This is true in our first patient who had multiple sexual partners and had a histologically proven presence of condyloma accuminatum, which has been shown to be associated with a 4-5 increased risk for penile cancer [8]. Even though anecdotal, it suffices to surmise that sexual promiscuity and uncircumcision played a significant role in carcinogenesis in the cases discussed. Smoking is also an established risk factor and although the exact mechanism is unknown, it has been suggested that the accumulation of nitrosamines in genital secretions could be the conduit for its development [2,8]. It is in this light that the smoking history in our second patient who admitted to smoking, spanning a period of 15 years is highly significant.

Partial penectomy with inguinal lymphadenopathy was the choice of treatment for both cases due to the advanced disease at presentation. As a result of vexing issues such as cultural beliefs, embarrassment, low self-esteem and the desire to seek alternative treatments first, most patients present in the advanced stages of the disease where cure is not guaranteed. Cassell *et al*. [2] report that most men in sub-Saharan Africa tend to report in the advanced stages of the disease portending poor prognosis. Another notable issue in the management of these patients is the psychological distress associated with the disease outcomes. Patients may be psychologically harmed by the partial amputation of their manhood which invariably affects their quality of life [13]. Available evidence suggest that patients enjoy good sexual life after penile preserving procedures such as chemotherapy and wide local excision, in contrast, there is a dip in sexual activity and quality of life after glansectomy and partial penectomy. As exemplified in our reported case, partial penectomy resulted in a considerable dip in sexual satisfaction and some dyspareunia although libido remained intact.

A review by Audenet and Sfakianos [13] reported a decrease in sexual function following partial penectomy with 66% of patients having reduced satisfaction postoperatively. In a separate study, D´Acona *et al*. [14] observed a 36% decline in sexual function post partial penectomy even though no significant levels of depressions and anxiety were reported. Our findings are therefore consistent with literature. With reference to quality of life, our reported case enjoys satisfactory quality of life with good performance in physical, role, emotional and social functioning. There was, however, a notable decline in cognitive function (50%) a year after surgery. Suarez-Ibarra *et al*. [15] reported similar findings of good outcomes on the assessed domains of quality of life; physical, emotional and role functions remained significantly normal. However, men treated with partial penectomy reported more pain compared to those treated with other modalities. It is quite clear that patients treated for penile cancer may notice a decline in sexual function and other functions of life. Although quality of life may be affected, the downturn is of low or moderate magnitude rather than high. It is therefore imperative that strong family and psychological support services are available before commencing treatment. The quality of life of patient and partner should be at the forefront of any treatment choice [2,13]. Although our study is limited by the number of cases reported, this is a very rare condition and the findings we believe, are very relevant for clinicians working within the sub region.

**Patient perspective:** our surviving patient enjoys satisfactory quality of life post treatment with physical and emotion functions being optimum. He however, reports of some dip in sexual function even though libido remains satisfactory.

**Informed consent:** patients voluntarily gave consent for their medical information and photographs to be published while maintaining anonymity.

## Conclusion

Penile cancer is a rare form of malignancy. The major risk factors as reported in this case study, include uncircumcision, previous treatment for sexually transmitted infections, multiple sexual partners and smoking. Treatment is associated with reduction in sexual function although quality of life may remain satisfactory. Strong family and psychological support are key cornerstones for good treatment outcomes.

## References

[1] Montes Cardona CE, Garcíía-perdomo HA (2018). Incidence of penile cancer worldwide: systematic review and meta-analysis. Rev Panam Salud Publica.

[2] Cassell A, Yunusa B, Manobah B, Wambo D (2020). Management guidelines of penile cancer-a contemporary review of sub-Saharan Africa. Infect Agent Cancer.

[3] Klufio GO (2004). A review of genitourinary cancers at the Korle Bu Teaching Hospital. West Afr J Med.

[4] Morrison BF (2014). Risk factors and prevalence of penile cancer. West Indian Med J.

[5] Marchionne E, Hui A, Perez C, Khachemoune A (2017). Penile squamous cell carcinoma: a review of the literature and case report treated with Mohs micrographic surgery. An Bras Dermatol.

[6] Kroon BK, Horenblas S, Nieweg OE (2005). Contemporary management of penile squamous cell carcinoma. J Surg Oncol.

[7] Heideman DAM, Waterboer T, Pawlita M, Delis-Van Diemen P, Nindl I, Leijte JA (2007). Human papillomavirus-16 is the predominant type etiologically involved in penile squamous cell carcinoma. J ClinOncol.

[8] Dillner J, von Krogh G, Horenblas S, Meijer CJ (2000). Etiology of squamous cell carcinoma of the penis. Scand J Urol Nephrol Suppl.

[9] Hakenberg OW, Dräger DL, Erbersdobler A, Naumann CM, Jünemann KP, Protzel C (2018). The diagnosis and treatment of penile cancer. Dtsch Arztebl Int.

[10] Hakenberg OW, Watkin N, Compérat E, Minhas S, Necchi A, Protzel C (2018). EAU guidelines penile cancer 2018. Association of Urology.

[11] Diorio GJ, Leone AR, Spiess PE (2016). Management of penile cancer. Urology.

[12] Morris BJ, Gray RH, Castellsague X, Bosch FX, Halperin DT, Waskett JH (2011). The strong protective effect of circumcision against cancer of the penis. Adv Urol.

[13] Audenet F, Sfakianos JP (2017). Psychosocial impact of penile carcinoma. Transl Androl Urol.

[14] D'Ancona CA, Botega NJ, De Moraes C, Lavoura NS, Santos JK, Rodrigues Netto N (1997). Quality of life after partial penectomy for penile carcinoma. Urology.

[15] Suarez-Ibarrola R, Cortes-Telles A, Miernik A (2018). Health-related quality of life and sexual function in patients treated for penile cancer. Urol Int.

